# Lemon protein disulfide isomerase: cDNA cloning and biochemical characterization

**DOI:** 10.1186/1999-3110-54-34

**Published:** 2013-09-16

**Authors:** Yu-Ting Chen, Lisa Wen, Kuo-Chuan Ho, Rong-Huay Juang, Chi-Tsai Lin

**Affiliations:** 1 Institute of Bioscience and Biotechnology and Center of Excellence for the Oceans, National Taiwan Ocean University, 2 Pei-Ning Rd, Keelung, 202 Taiwan; 2 Institute of Genomics and Bioinformatics, Agricultural Biotechnology Center, National Chung Hsing University, Taichung, Taiwan; 3 Department of Chemistry, Western Illinois University, 1 University Circle, Macomb, IL 61455-1390 USA; 4 Department of Biochemical Science and Technology, National Taiwan University, Taipei, 106 Taiwan

**Keywords:** *Citrus limonum*, Protein disulfide isomerase (PDI), Scrambled RNase A (sRNase A), Three-dimension structural model (3-D structural model)

## Abstract

**Background:**

Protein disulfide isomerases (PDIs), a family of structurally related enzymes, aid in protein folding by catalyzing disulfide bonds formation, breakage, or isomerization in newly synthesized proteins and thus.

**Results:**

A ClPDI cDNA (1828 bp, GenBank accession HM641784) encoding a putative PDI from *Citrus limonum* was cloned by polymerase chain reaction (PCR). The DNA sequence encodes a protein of 500 amino acids with a calculated molecular mass of 60.5 kDa. The deduced amino acid sequence is conserved among the reported PDIs. A 3-D structural model of the ClPDI has been created based on the known crystal structure of *Homo sapiens* (PDB ID: 3F8U_A). The enzyme has two putative active sites comprising the redox-active disulfides between residues 60–63 and 405–408 (motif CGHC). To further characterize the ClPDI, the coding region was subcloned into an expression vector pET-20b (+), transformed into *E. coli* Rosetta (DE3)pLysS, and recombinant protein expressed. The recombinant ClPDI was purified by a nickel Sepharose column. PDI’s activity was assayed based on the ability of the enzyme to isomerize scrambled RNase A (sRNase A) to active enzyme. The *K*_M_*, k*_cat_ and *k*_cat_/*K*_M_ values were 8.3 × 10^-3^ μM, 3.0 × 10^-5^ min^-1^, and 3.6 × 10^-1^ min^-1^ mM^-1^. The enzyme was most active at pH 8.

**Conclusions:**

The advantage of this enzyme over the PDI from all other sources is its low *K*_M_. The potential applications of this PDI in health and beauty may worth pursuing.

**Electronic supplementary material:**

The online version of this article (doi:10.1186/1999-3110-54-34) contains supplementary material, which is available to authorized users.

## Background

Protein disulfide isomerase (PDI), a member of thioredoxin-superfamily, plays a key role in catalyzing disulfide bond formation, reduction, and isomerization. It was the first reported protein folding enzyme (Venetianer and Straub [Bibr CR20][Bibr CR21]; Goldberger et al. 3^1963^). PDI is best known as resided in endoplasmic reticulum (ER) where folding, modification, and quality control of many secretory and cell-surface proteins take place (Hatahet and Ruddock 2009; Lambert and Freedman 1983). *In vivo*, native disulfide bond formation involves the formation of new disulfides (oxidation) and the rearrangement of non-native disulfides (isomerization). PDI, contains combinations of catalytically active and inactive thioredoxin domains, is capable of catalyzing both the oxidation of new disulfides and the isomerization of existing disulfides (Kulp et al. 2006). The first and last domains (referred to as a and a’ domain, respectively) contain CxxC active site motifs, whereas the two middle domains (referred to as b and b’ domain) are catalytically inactive (Kemmink et al. 1997). Oxidation, catalyzed by the a’ domain, involves the transfer of an active site disulfide from PDI to substrate proteins. Isomerization, catalyzed by the a domain, requires the active site cysteines to be in the reduced form in order to attack non-native disulfides in substrate proteins thereby catalyzing their rearrangement (Kulp et al. 2006).

Like other proteins with thioredoxin folds, PDI is a multifunctional protein (Pedone et al. 2010). In addition to catalyze protein folding, PDI is the β-subunit of prolyl-4-hydroxylase (Koivu et al. 1987), is a subunit of microsomal triglyceride transfer protein (Wetterau et al. 1991), acts as a chaperone (Wang and Tsou 1993), is implicated in peptide loading onto MHC class I (Peaper and Cresswell 2008), and is involved in regulating NAD(P)H oxidase (Janiszewski et al. 2005). Although members of PDI enzyme have a classic ‘KDEL’ ER retention signal at the C-terminal end (Pelham 1990), the enzymes have been identified outside the ER at many subcellular locations and are known to involve in a wide range of biological functions (Turano et al. 2002). Outside of ER, PDI has been reported to serve as switches for modulating protein function in some cases (Hogg 2003; Wouters et al. 2010). For instance, extracellular PDI mediated disulfide exchange has been shown to switch tissue factor between coagulation to cell signaling (Wouters et al. 2010; Ahamed et al. 2006).

Lemon is an economically valuable produce in Taiwan. It is an amazing fruit that has been used as natural remedy for health and beauty. PDI has been used as an ingredient in cosmetic agents for treating hair (European Patent WO2003099243 A1). Here, we report the cloning of a putative PDI cDNA from lemon, namely ClPDI. The coding region of the ClPDI cDNA was introduced into an *E. coli* expression system and the active enzyme purified and characterized. Understanding the properties of this ClPDI will be beneficial for its potential applications such as catalyzing protein folding or serving a chaperone contributing to reactivation of inactive enzymes.

## Methods

### Total RNA preparation from lemon and cDNA synthesis

A fresh lemon (*Citrus limonum*) was obtained from a local market. The lemon including the skin (4 g) was frozen in liquid nitrogen and ground to powder in a ceramic mortar. PolyA mRNA (35 μg) was prepared using Straight A’s mRNA Isolation System (Novagen, USA). Four μg of the mRNA was used in the 5′-RACE-Ready cDNA and 3′-RACE-Ready cDNA synthesis using Clontech’s SMART RACE cDNA Amplification Kit.

### Isolation of ClPDI cDNA

Using the 5′-RACE-Ready cDNA of lemon as a template and two degenerate primers (5′ AGY CAA GGT GCH TTC CAG 3′ and ACY TTM ACW GGC TCR TTG TT), a 0.2 kb fragment was amplified by PCR. The degenerate primers were designed based on the conserved sequences of PDI from AtPDI (*Arabidopsis thaliana*, AY063059), HsPDI (*Homo sapiens*, 3F8U_A), MmPDI (*Mus musculus*, 2DJ2_A), HiPDI (*Humicola insolens*, 2DJJ_A). The 0.2 kb fragment was subcloned and sequenced. On the basis of this DNA sequence, two primers near both ends, a ClPDI-6R primer (5′ ACY TTM ACW GGC TCR TTG TT 3′) and a ClPDI-7 F primer (5′ GCA CCT TGG GTG AAG GAA TAC 3′) were synthesized. The primers allowed sequence extension from both ends of the 0.2 kb fragment when used with the UPM primer (universal primer A mix, purchased from BD biosciences). Two PCRs were carried out each using 0.1 μg of the 5′-RACE-Ready cDNA or 3′-RACE-Ready cDNA as a template. The primer pairs in each reaction were UPM and ClPDI-6R primers, and UPM and ClPDI-7 F primers. A 1.3 kb fragment (5′-RACE; 5′-DNA end) and a 1.0 kb DNA (3′-RACE; 3′-DNA end) were amplified by PCR. Both DNA fragments were subcloned into pCR4 vector and transformed into *E. coli* TOPO10. The nucleotide sequences of these inserts were determined in both strands. Sequence analysis revealed that the combined sequences covered an open reading frame of a putative ClPDI cDNA (1828 bp, EMBL no. HM641784). The identity of this ClPDI clone was assigned by comparing the inferred amino acid sequence in various data banks using the basic local alignment search tool (BLAST).

### Bioinformatics analysis of ClPDI

The BLASTP program was used to search homologous protein sequences in the nonredundant database (NRDB) at the National Center for Biotechnology Information, National Institutes of Health (http://www.ncbi.nlm.nih.gov/). Multiple alignments were constructed using ClustalW2 program. Protein secondary structure was predicted by SWISS-MODEL program and represented as α helices and β strands. A 3-D structural model of PDI was created by SWISS-MODEL (http://swissmodel.expasy.org/) based on the known crystal structure of *Homo sapiens* PDI (PDB code 3F8U_A) (Dong et al. 2009). The modeling data was then superimposed with that of PDB ID: 3F8U_A by DeepView Swiss-PdbViewer v4.1 (http://spdbv.vital-it.ch/).

### Subcloning of ClPDI cDNA into an expression vector

The coding region of the ClPDI cDNA was amplified using gene specific flanking primers. The 5′ upstream primer contains *Eco* RI recognition site (5′ CGT CTC GAA TTC GAT GGC CAG TCG ATC GAT 3′) and the 3′ downstream primer contains *Not* I recognition site (5′ GCG GCC GCG AGC TCA TCT TTT CCA GA 3′). Using 0.2 μg of ClPDI cDNA as a template, and 10 pmole of each 5′ upstream and 3′ downstream primer, a 1.5 kb fragment was amplified by PCR. The fragment was ligated into pCR4 and transformed into *E. coli*. The recombinant plasmid was isolated and double digested with *Eco* RI and *Not* I. The digestion products were separated on a 1.0% agarose gel. The 1.5 kb fragment was gel purified and subcloned into the *Eco* RI and *Not* I site of pET-20b(+) expression vector (Novagen). The recombinant DNA was then transformed into *E. coli* Rosetta (DE3)pLysS and protein expressed by isopropyl β-D-thiogalactopyranoside (IPTG) induction. Expression of functional recombinant protein was demonstrated by enzyme activity assay as described below.

### Expression and purification of the recombinant ClPDI

The transformed *E. coli* containing the ClPDI gene was grown at 37°C in 200 mL of Luria-Bertani containing 50 μg/mL ampicillin until *A*_*600*_ reached 0.6. Protein expression was induced by the addition of IPTG to a final concentration of 50 μM. The culture was incubated at 80 rpm for an additional 2 h at 32°C. The cells were harvested and soluble proteins extracted in PBS with glass beads as described before (Ken et al. 2005). The recombinant ClPDI was purified by Ni-NTA affinity chromatography (elution buffer: 30% PBS containing 20–250 mM imidazole) as per the manufacture’s instruction (Qiagen). The purified protein was checked by a 10% SDS-PAGE. Protein bands on gel were visualized by staining with Coomassie Brilliant Blue R-250. Protein concentration was determined by a Bio-Rad Protein Assay Kit (Richmond, CA) using bovine serum albumin as a reference standard.

### Molecular mass analysis via electrospray ionization quadrupole-time-of-flight (ESI Q-TOF)

The purified recombinant ClPDI (0.21 mg/mL) was prepared in 0.1 mM Tris–HCl containing 0.05 mM NaCl, 0.5 mM imidazole and 0.03% glycerol. The sample (5 μL) was used for molecular mass determination using an ESI Q-TOF mass spectrometer (Micromass, Manchester, England).

### ClPDI activity assay

PDI activity was assayed by the method of Ibbetson and Freedman (1976) using scrambled ribonuclease (sRNase A) as a substrate. In this method, PDI was used to activate sRNase A then the ribonuclease activity was monitored spectrophotometriclly as described below. Each 5 μL ClPDI sample (0.2 μg/μL stock solution) was first pre-incubated with 10 μL of 100 μM DTT (dithiothreitol) for 5 min at 25°C. Next, a 0–60 μL portion of the sRNase A (0.036 μM) was added, followed by addition of 10 μL of 0.5 M Tris/HCl, pH 7.5 and appropriate amount of H_2_O to 90 μL. The mixture was incubated for 20 min at 25°C to allow conversion of sRNase A to active RNase by PDI. The RNase activity was then measured by its ability to degrade RNA. Ten μL of RNA (2 μg/μL) was added to each assay mixture (Total volume was 100 μL). The samples were incubated at 37°C for another 5 min. Three hundred μL of 95% ethanol was added to each assay mixture to precipitate the residual RNA. Ribonuclease activity was monitored by observing the decrease in *A*_*260*_ of the residual RNA.

### Kinetic studies

The kinetic properties of the ClPDI (1.0 μg) was determined by varying the concentrations of sRNase A (3.6 to 21.6 nM) with fixed amount of 20 μg RNA (2 μg/μL). The change in absorbance at 260 nm was recorded between 1 and 20 min. The *K*_M_, V_max_ and *k*_cat_ were calculated from Lineweaver-Burk plots.

### Enzyme characterization

The ClPDI enzyme was tested for stability in terms of its activity under various conditions. Aliquots of the CIPDI sample (1.0 μg) were treated as follows: (1) *Thermal effect*. Each enzyme sample (1.0 μg) was heated at 37, 50, 60, 70, or 80°C for 20 min. (2) *pH effect*. Each enzyme sample (1.0 μg) was adjusted to desired pH by adding a half volume of buffer with different pHs: 0.2 M citrate buffer (pH 2.5, or 4.0), 0.2 M phosphate buffer (pH 6.0, 7.0 or 8.0) or 0.2 M CAPS buffer (pH 10.0, or 11.0). Each sample was incubated at 37°C for 1 h. (3) *Imidazole effect*. During protein purification, the CIPDI enzyme was eluted with imidazole, therefore, its effect on activity was examined. Imidazole was added to each enzyme sample to the final levels of 0.2, 0.4, 0.8 or 1.0 M and incubated at 37°C for 1 h. (4) *DTT effect.* DTT was added to each enzyme sample to the final levels of 10, 30, 70, 100 or 200 μM and incubated at 37°C for 5 min. At the end of each treatment, samples were checked for CIPDI activity.

## Results and discussion

### Cloning and characterization of a cDNA encoding ClPDI

**Figure 1:**
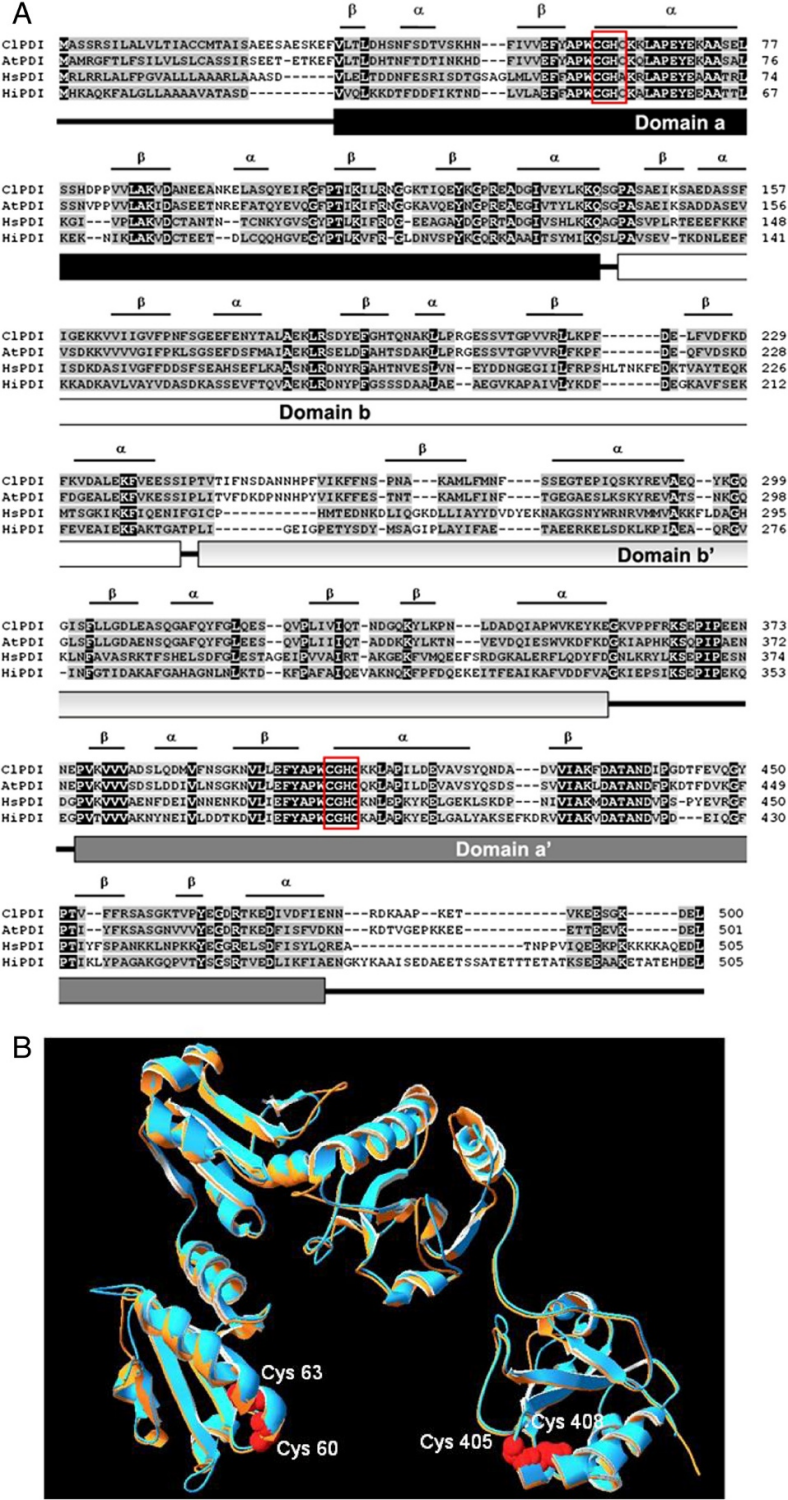
**Alignment of the amino acid sequences of ClPDI with other organism’s PDI and 3-D structural model. (A)** Sequence alignment: ClPDI (this study), AtPDI (*Arabidopsis thaliana*, AY063059), HsPDI (*Homo sapiens*, P30101), HiPDI (*Humicola insolens*, AAC60578). Identical amino acids in all sequences are shaded black, conservative replacements are shaded gray. Two red boxes denote highly conserved catalytic motifs (C^60^GHC^63^, C^405^GHC^408^). Protein secondary structure was predicted by SWISS-MODEL program and represented as α helices and β strands. Underline shows the positions of the domains corresponding to domains a, b, b’ and a’. **(B)** A 3-D structural model of ClPDI. The structural model of the ClPDI was created based on the known crystal structure of *Homo sapiens* PDI (PDB: 3F8U_A) via SWISS-MODEL program and was superimposed with PDB ID: 3F8U_A (orange) to obtain structure alignment via SPDBV_4 program.

A putative ClPDI cDNA clone was identified on the basis of the consensus pattern and sequence homology to other published PDIs in NCBI database. The entire coding region of ClPDI cDNA is 1503 bp and the deduced protein consists of 500 amino acid residues with a calculated molecular mass of 60.5 kDa (Accession no. HM641784). Figure 1 shows the optimal alignment of the amino acid sequences of the ClPDI with 3 selected ClPDI sequences from other sources. This ClPDI shared 70% identity with AtPDI (*Arabidopsis thaliana*, AY063059), 35% with HsERp57 (*Homo sapiens*, NP_005304, 3F8U_A), and 37% with HiPDI (*Humicola insolens*, AAC60578, 2DJJ_A). The two highly conserved catalytic motifs are denoted in red boxes (C^60^GHC^63^, C^405^GHC^408^, Figure 1A) located in the two putative catalytic domains, a and a’. The secondary structure, predicted by SWISS-MODEL program, showed 14 α helices and 19 β strands. The structure of PDI is currently recognized as having four distinct domains, a, b, b’, and a’ (as marked in Figure 1A), plus a highly acidic C-terminal extension (which contains the ER-localization motif: KDEL) and an interdomain linker between the b’ and a’ domains (Hatahet and Ruddock 2009; Wang et al. 2010). The 3-D structural model was superimposed with PDB ID: 3F8U_A (orange) via the SPDBV_4 program was shown using protein solid ribbon (Figure 1B).

### Expression and purification of the recombinant ClPDI

**Figure 2:**
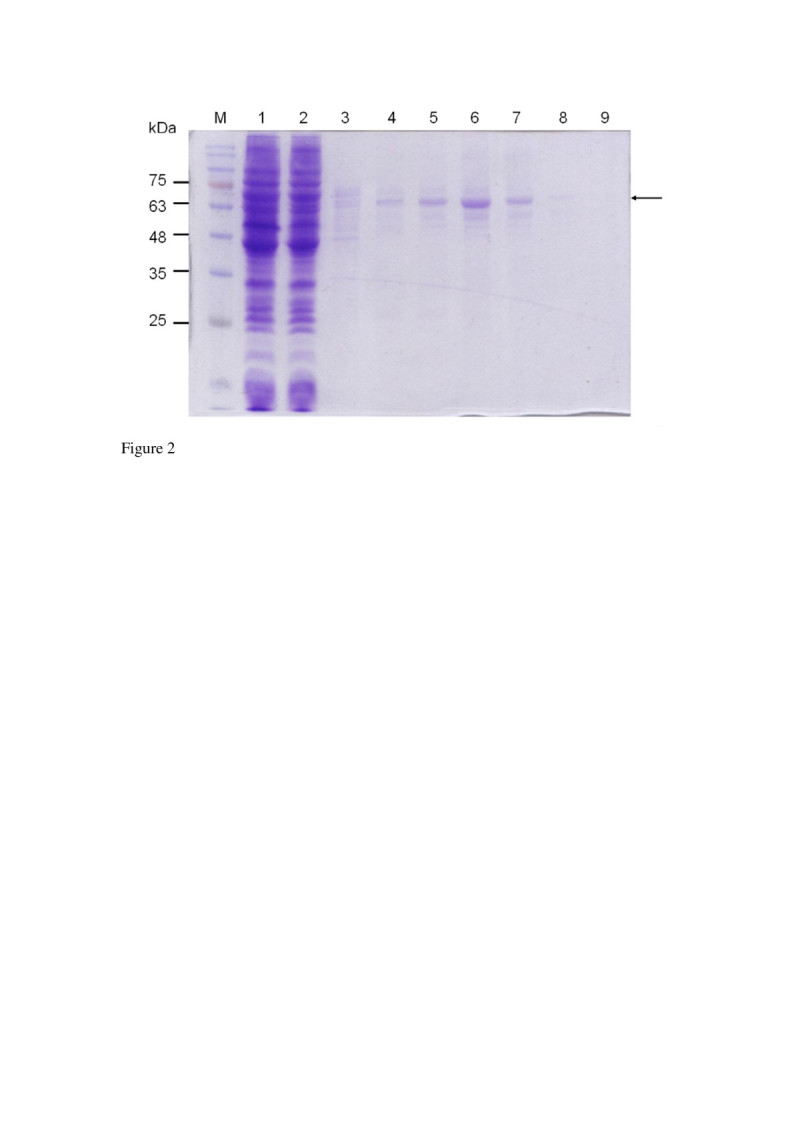
**Expression and purification of recombinant ClPDI in** ***E. coli.*** Fifteen μL (loading buffer without mercaptoethanol and without boiling) of each fraction was loaded into each lane of the 10% SDS-PAGE followed by Coomassie Brilliant Blue R-250 staining. Lane 1, crude extract from *E. coli* expressing ClPDI; 2, flow-through proteins from the Ni-NTA column; 3, wash; 4–7, ClPDI eluted from Ni-NTA column. Molecular masses (in kDa) of standards are shown at left.

The coding region of the ClPDI (1503 bp) was amplified by PCR and subcloned into an expression vector, pET-20b (+) as described in the Materials and Methods. Positive clones were verified by DNA sequence analysis. The recombinant ClPDI was expressed, and the proteins were analyzed by a 10% SDS-PAGE in the absence of reducing agent and without boiling (Figure 2). The recombinant ClPDI was not expressed in the absence of IPTG (data not shown). In the presence of IPTG, it was expressed as a His_6_-tagged fusion protein and was purified by affinity chromatography with nickel chelating Sepharose. A major band of ~63 kDa (expected size of recombinant ClDPI monomer) was detected in Ni-NTA eluted fractions by SDS-PAGE (Figure 2, lanes 5–7). The fractions contained pure protein were pooled and characterized further. Analysis of the ClPDI by ESI Q-TOF confirms the presence of a single protein with molecular mass of 60.5 kDa. This indicates that the enzyme is predominantly monomeric in nature. The yield of the purified His_6_-tagged ClPDI was 875 μg from 100 mL of culture. Functional ClPDI was detected by activity assay as describe below.

### Kinetic studies of the purified ClPDI

**Figure 3:**
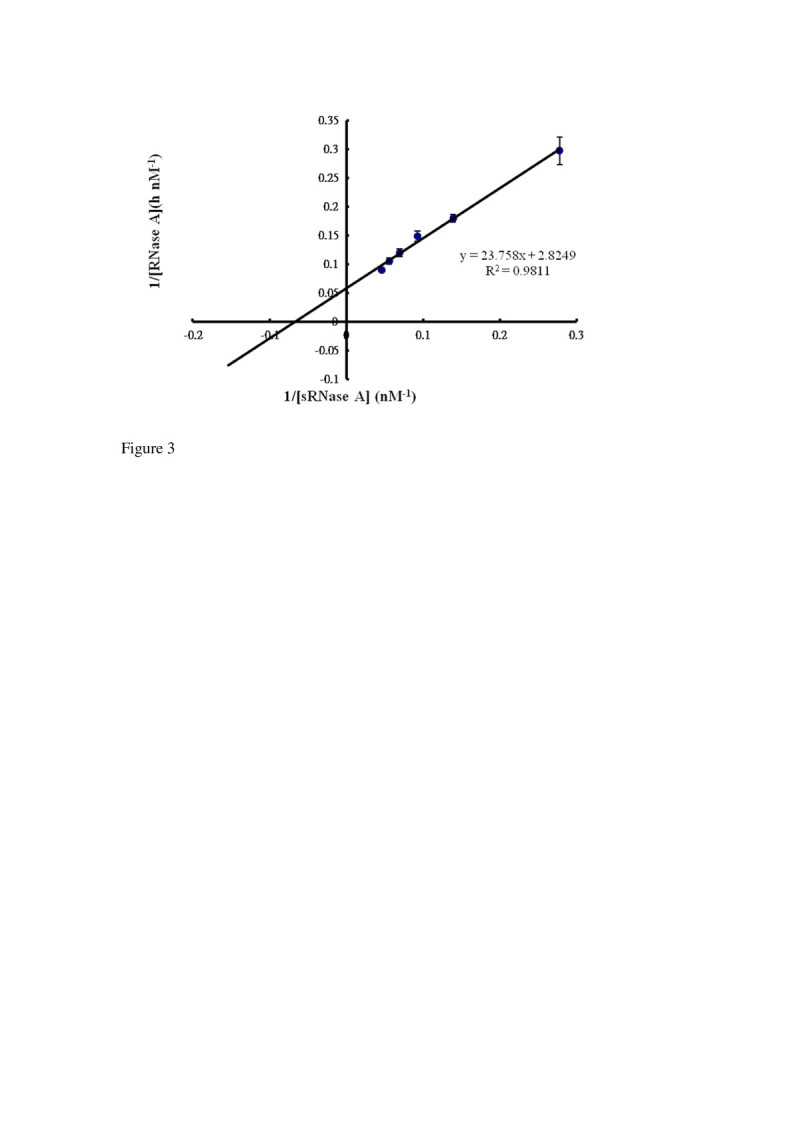
**Double-reciprocal plot of varying ‘scrambled’ ribonuclease A (sRNase A) on ClPDI activity.** The initial rate of the enzymatic reaction was measured with change in absorbance at 260 nm between 1 and 20 min at 20 μg RNA with the sRNase A varied from 3.6 to 21.6 nM. The *K*_M_*, k*_cat_ and *k*_cat_/*K*_M_ were calculated from Lineweaver-Burk plots.

The purified recombinant CIPDI possesses PDI activity as demonstrated by its ability to activate sRNase A through oxidation and shuffling of disulfide bonds. Kinetic study of the CIPDI was done by varying substrate concentration of sRNase A. As shown in Figure 3, the Lineweaver-Burk plot of the velocity (1/V) against 1/sRNAase A gave the *K*_M_*, k*_cat_ and *k*_cat_/*K*_M_ values were 8.3 × 10^-3^ μM, 3.0 × 10^-5^ min^-1^, 3.6 × 10^-1^ min^-1^ mM^-1^. Comparison of the *K*_M_ with that of PDI from other available sources (Table 1) reveals that lemon’s *K*_M_ is several orders of magnitude smaller. The result indicating that the ClPDI can work under extremely low substrate concentration. The wide variation of *K*_M_ values among the reported data may due to differences in reaction conditions and whether the redox buffer was sufficiently reducing to maintain PDI in an active form. According to Lambert and Freedman (1983) that the bovine liver PDI requires the presence of either a dithiol or a thiol. Dithiothreitol is effective at concentrations 100-fold lower than that of monothiols such as reduced glutathione or cysteamine. The enzyme follows Michaelis-Menten kinetics with respect to these substrates; *K*_M_ values were 4, 620 in the presence of reduced glutathione and 380 μM in the presence of DTT. This is one reason to say why animal’s (bovine, and rat) *K*_M_ values (8 to 380 μM) vary so widely. This reason was also supported by Lyles and Gilbert (1991) that the rat PDI’s catalysis depends on the composition of the redox buffer. The human PDI was assayed using fluorescence-quenched peptides as substrate instead of sRNase A. Therefore, a direct comparison cannot be made.

**Table 1:** **Kinetic characterization of ClPDI and that from other sources**

Species	***K***_M_	***k***_cat_	***k***_cat_/***K***_M_	Ref.
	(μM)	(min^-1^)	(min^-1^ mM^-1^)	
Lemon	8.3 × 10^-3^	3.0 × 10^-5^	3.6 × 10^-1^	This study
Human*	0.21 ~ 2	0.21 ~ 0.89	1 ~ 0.44	Westphal et al. 1998
Bovine	8.0 ± 1.5	0.46 ± 0.05	5.58 × 10^-2^	Lyles and Gilbert 1991
Bovine	380	NA	NA	Lambert and Freedman 1983
Rat	67 ± 7	1.3 ± 0.1	1.94 × 10^-2^	Walker and Gilbert 1997

Values for PDI are from this work (lemon) or from the literatures (human, bovine, and rat ).

*Human PDI was assayed using fluorescence-quenched peptides as substrate instead of sRNase A.

The *k*_cat_ value of ClPDI is also several orders of magnitude smaller than that of PDI from other sources (Table 1). But its *k*_cat_/*K*_M_ value is compatible to that of PDI from other sources. It is likely that the ClPDI is one of the early enzymes that responsible for oxidative folding of proteins in lemon as the enzyme can respond to extremely low substrate concentration.

### Characterization of the purified ClPDI

**Figure 4:**
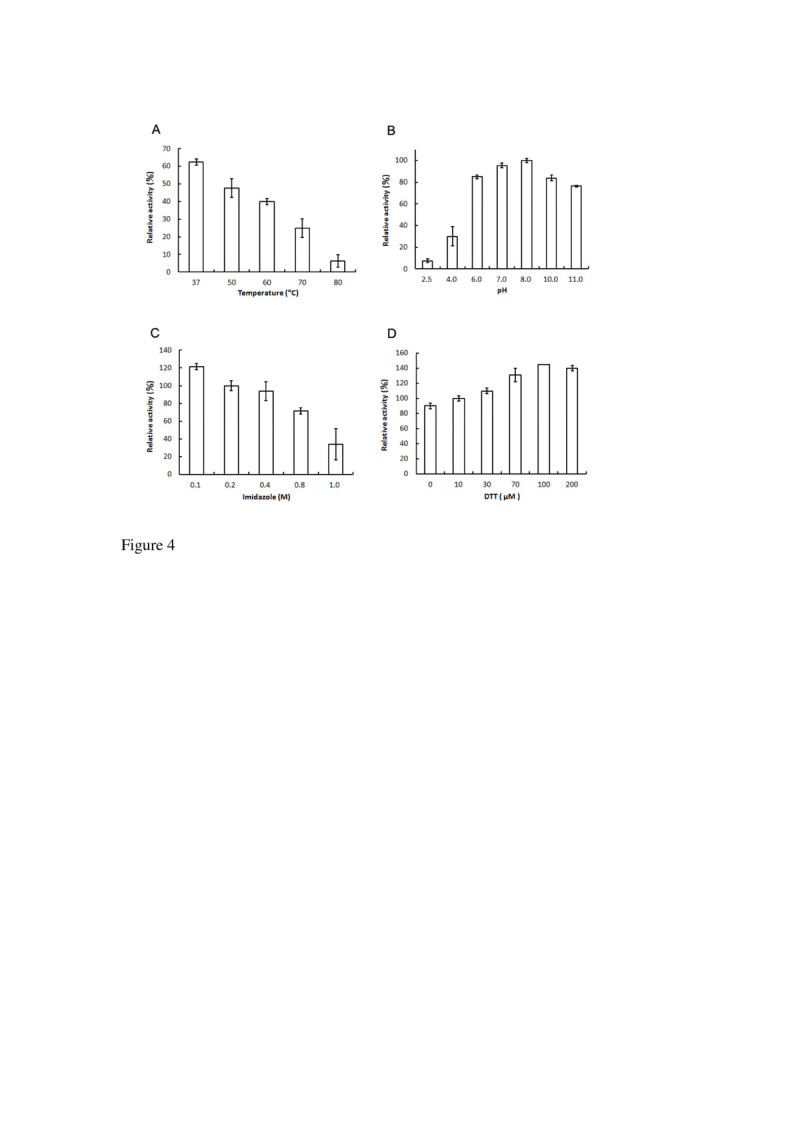
**Effect of temperature, pH, imidazole, and DTT on the purified ClPDI.** The enzyme sample (1.0 μg) was heated at 37, 50, 60, 70, or 80°C for 20 min. At the end of each treatment, samples were assayed for CIPDI activity at pH 7.5 **(A)**. Aliqouts of the enzyme sample were incubated with different pH buffers at 37°C for 1 h and then assayed for CIPDI activity **(B)**. Imidazole was added to each enzyme sample (1.0 μg) to the final levels of 0.2, 0.4, 0.8 or 1.0 M and incubated at 37°C for 1 h and then assayed for CIPDI activity at pH 7.5 **(C)**. DTT was added to each enzyme sample (1.0 μg) to the final levels of 10, 30, 70, 100 or 200 μM and incubated at 37°C for 5 min and then assayed for CIPDI activity at pH 7.5 **(D)**. Data are means of three experiments.

The stability of the enzyme activity was characterized under various conditions. As shown in Figure 4A, thermal stability of the ClPDI was tested to examine the effect of heat on the PDI activity. The purified ClPDI was heat-treated as described in the Materials and Methods and then analyzed for the residue PDI activity. A control reaction where the enzyme was treated at 25°C was counted as 100% activity. The enzyme activity decreased as the temperature increased. There was only 8% detectable activity when the enzyme was heated at 80°C for 20 min. In Figure 4B, the ClPDI is activity under a broad pH range from 6–11 with an optimal activity at pH 8.0. The enzyme retained 30% activity at pH 4.0. The enzyme showed a decrease in its activity with increasing imidazole concentration from 0.2-1 M (Figure 4C). Approximately 70% activity was lost in the presence of 1 M imidazole. The enzyme activity was enhanced in the presence of thiol reducing agent (DTT), the ClPDI activity was correlated with the increasing concentration of DTT from 10 to 100 μM (Figure 4D) and it reached the plateau at approximately 100 μM.

## Conclusion

The importance of PDI has been implicated in health and disease (Benham 2012; Andreu et al. 2012). This study reported the first cloning and expression of an important protein folding enzyme, ClPDI, from lemon. The active form of the ClPDI has been successfully expressed in *E. coli* and characterized. The advantage of this enzyme over the PDI from all other sources is its extremely low *K*_M_. The potential applications of this enzyme in health and beauty may worth pursuing.

## Authors’ original submitted files for images

Below are the links to the authors’ original submitted files for images. Authors’ original file for figure 1 Authors’ original file for figure 2 Authors’ original file for figure 3 Authors’ original file for figure 4

## Abbreviations

PDI: Protein disulfide isomerase
sRNase A: Scrambled RNase A
IPTG: Isopropyl β-D-thiogalactopyranoside
SDS-PAGE: Sodium dodecyl sulfate-polyacrylamide gel electrophoresis
PBS: Phosphate buffer saline.
