# Supplementary figures and images for: Lemon protein disulfide isomerase: cDNA cloning and biochemical characterization
Source: Bot Stud. 2013 Sep 16;54:34. doi: 10.1186/1999-3110-54-34 (PMC5432843; doi:10.1186/1999-3110-54-34)

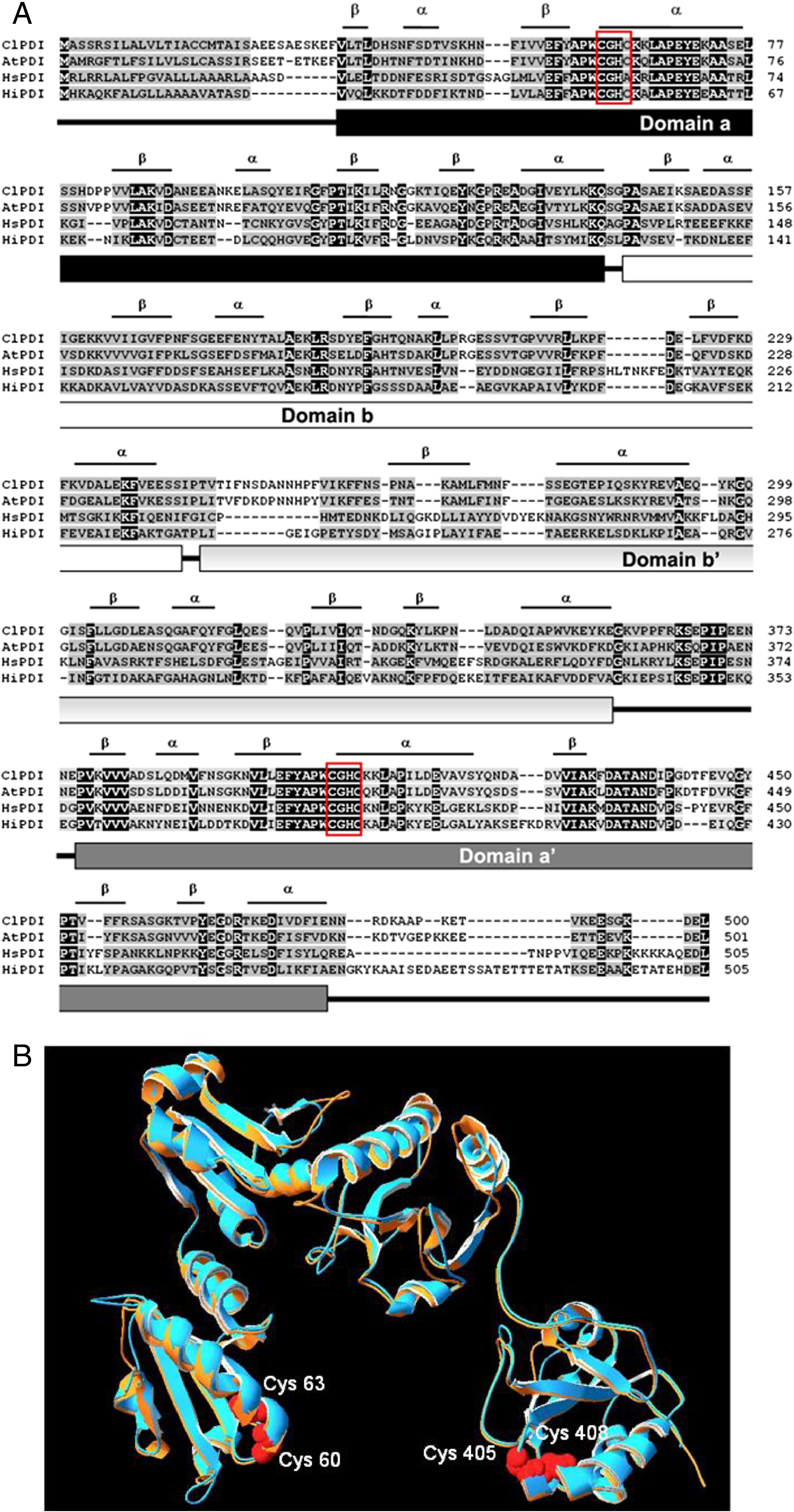

Supplement: Supplementary file 1 — Authors’ original file for figure 1 [file 40529_2013_28_MOESM1_ESM.tif]

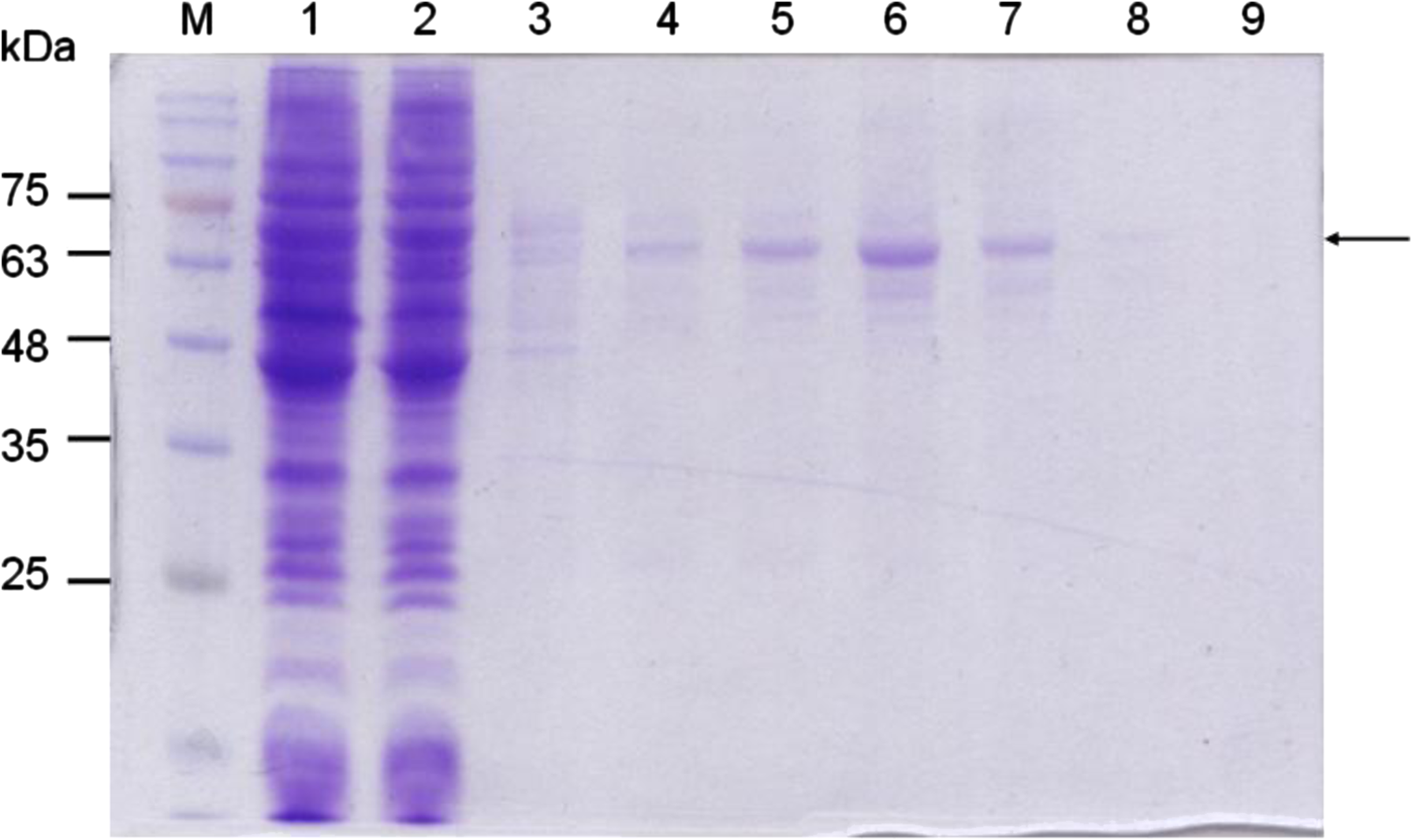

Supplement: Supplementary file 2 — Authors’ original file for figure 2 [file 40529_2013_28_MOESM2_ESM.tif]

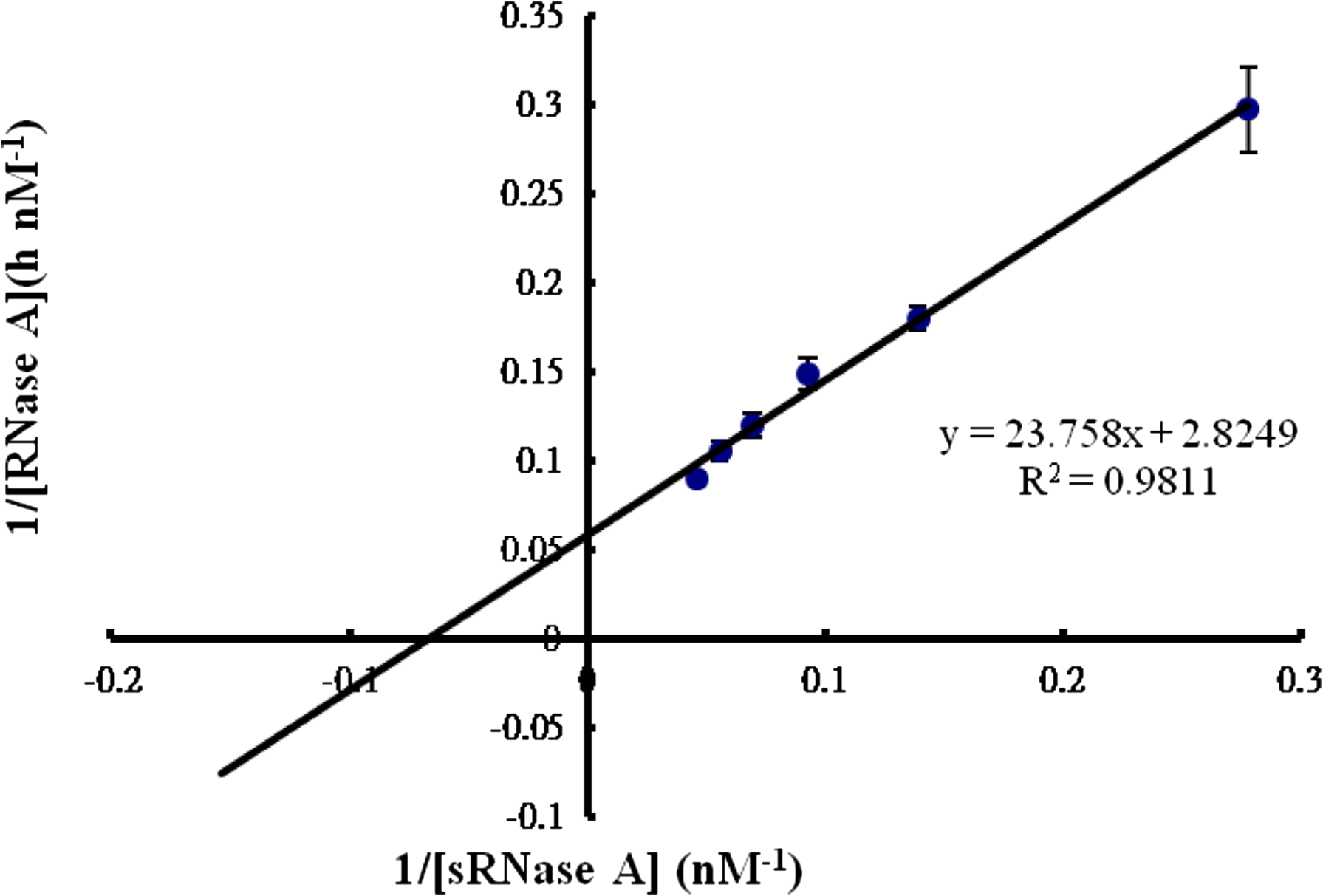

Supplement: Supplementary file 3 — Authors’ original file for figure 3 [file 40529_2013_28_MOESM3_ESM.tif]

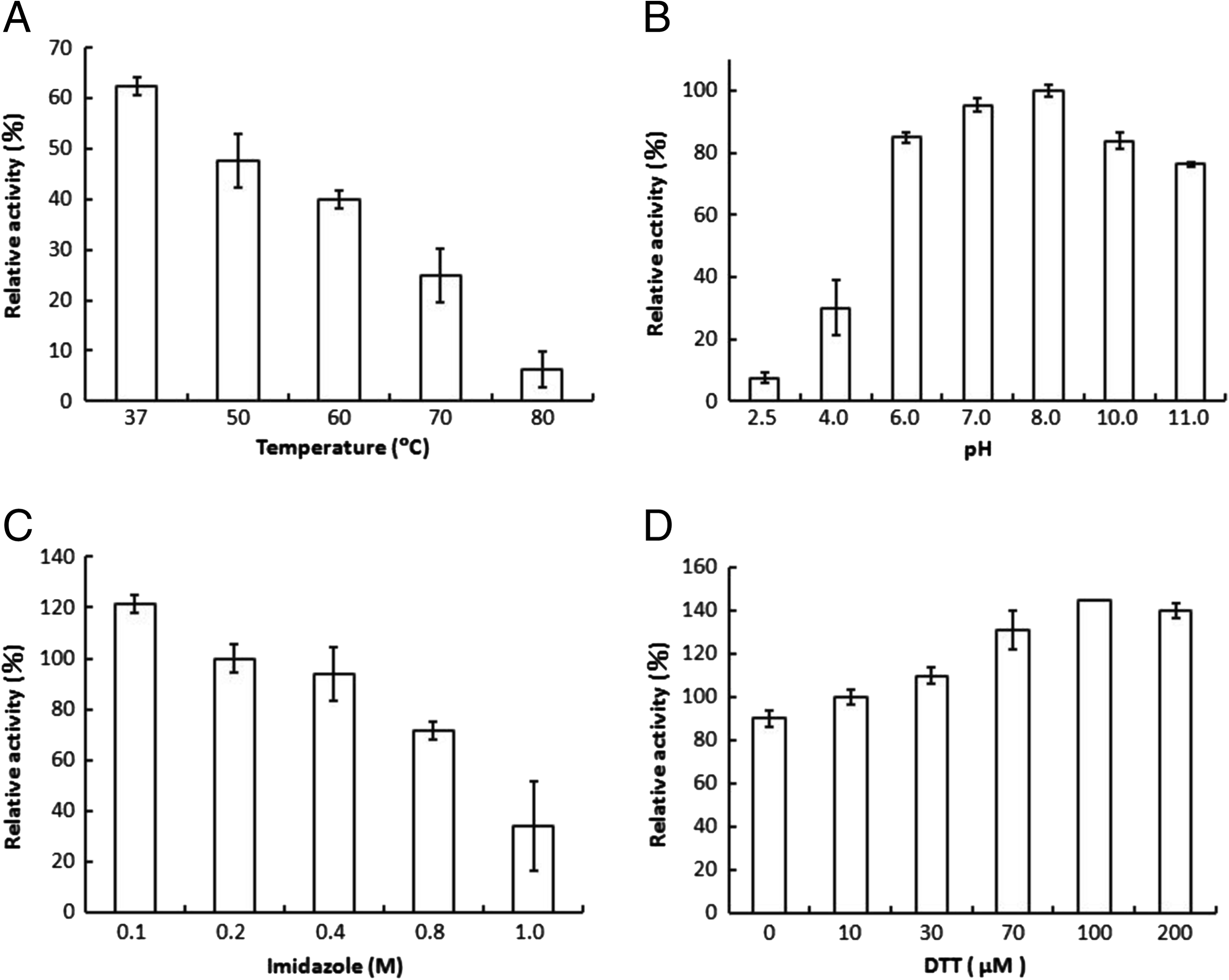

Supplement: Supplementary file 4 — Authors’ original file for figure 4 [file 40529_2013_28_MOESM4_ESM.tif]
